# Analysis of Volatile Compounds in *Jinhua ham* Using Three Extraction Methods Combined with Gas Chromatography–Time-of-Flight Mass Spectrometry

**DOI:** 10.3390/foods11233897

**Published:** 2022-12-02

**Authors:** Dengyong Liu, Cong Yang, Lu Bai, Xi Feng, Yanping Chen, Yin Zhang, Yuan Liu

**Affiliations:** 1 College of Food Science and Technology, Bohai University, Jinzhou 121013, China; 2 Department of Food Science & Technology, School of Agriculture & Biology, Shanghai Jiaotong University, Shanghai 200240, China; 3 Department of Nutrition, Food Science and Packaging, San Jose State University, San Jose, CA 95192, USA; 4 Key Laboratory of Meat Processing of Sichuan, Chengdu University, Chengdu 610106, China

**Keywords:** *Jinhua ham*, SAFE, SPME, NT, GC–TOF/MS, volatiles

## Abstract

The volatile compounds in *Jinhua ham* samples after different aging times were characterized using solvent-assisted flavor evaporation (SAFE), solid-phase microextraction (SPME), and needle trap (NT) extraction methods combined with gas chromatography–time-of-flight mass spectrometry (GC–TOF/MS). Hundreds of aroma compounds were identified, including aldehydes, alcohols, ketones, furans, esters, acids, pyrazines, and sulfides. The results showed that NT extracted the greatest number of volatile compounds, whereas the extraction efficiency of SPME headspace adsorption was highest among the three sample preparation methods. Principal component analysis of SPME effectively distinguished the variation in the aroma of the *Jinhua hams* specific to aging time. Butyrolactone, 2,6-dimethylpyrazine, 2,3,5-trimethylpyrazine, phenylacetaldehyde, and acetic acid were considered as the main volatile compounds in the *Jinhua ham* samples at three years of aging. The results showed that SPME–GC–TOF/MS effectively discriminated among samples by age. By comparing the three extraction methods, this study provides a theoretical basis for the selection of extraction methods of volatile aroma compounds in *Jinhua ham*.

## 1. Introduction

*Jinhua ham* is a traditional Chinese dry-cured meat product, and it has been standardized and industrialized in recent years. *Jinhua ham* is made from pig legs by salting, washing, sun-drying, shaping, ripening, and post-ripening. These processes provide the ham with a unique flavor [1]. Significant research progress has been made in elucidating the major volatile compounds and mechanisms of flavor formation in dry-cured meat products. The main aroma components of *Jinhua ham* are octanol, 2-methylbutanol, butanone, 2-hexanone, 2-heptanone, acetoin, γ-butyrolactone, butanal, 3-methylbutyraldehyde, propyl acetate, and 3-methylbutanoic acid [2]. However, extraction methods are often complex, expensive, and time-consuming. It is necessary to develop a fast, reliable, and effective method to analyze the volatile profiles of dry-cured ham products that can separate hundreds of volatile compounds in samples and identify the key aroma compounds that contribute to the overall aroma.

Solvent-assisted flavor evaporation (SAFE) utilizes high-vacuum distillation to isolate volatile compounds from the matrix at low temperature, thus avoiding potential flavor modification or artifacts due to the formation of volatile compounds when heated [3]. Therefore, SAFE can be used to extract low-boiling-point and heat-sensitive components and has been widely used in the flavor extraction of milk, oil, dry-cured ham, etc. [4,5]. However, there are also some disadvantages to SAFE. The extraction takes a long time, requires the addition of antifoaming agents to the sample, and uses organic solvents. In addition, the glassware and pumps are expensive and difficult to clean.

Solid-phase microextraction (SPME) technology is frequently used because of its simplicity, rapidity, lack of reagents, high sensitivity, small sample size, and lower cost characteristics [6,7]. Garcia-Esteban et al. [8] used SPME and simultaneous distillation extraction technology to analyze the volatile compounds in dry-cured ham. The analytes were concentrated on a fused silica fiber coating from the headspace air of the sample bottle. Although SPME technology has many advantages, it also has limitations: the coating fiber needs to be activated before use and is fragile and easy to damage. When isolating analytes, SPME is based on the equilibrium between the sample matrix and the immobilized sorbent, hence the calibration is complicated. This extraction can vary according to the polarity of the extracting fiber and sample amounts, as well as the time and the temperature of extraction, which all directly impact the efficiency of the process. In addition, SPME is more sensitive to volatile and semi-volatile compounds due to its lower extraction temperature.

Needle trap (NT) extraction is a simple, efficient, and environmentally friendly technique that integrates sampling, extraction, concentration, and injection, and it is suitable for sampling and analysis of trace organic components [9]. The NT device uses a specially designed stainless-steel needle packed with a suitable sorbent material as the extraction medium. Extraction is performed by drawing the sample inside the needle through the sorbent bed. During this process, the analytes are trapped on the sorbent material and then they are subjected to thermal desorption [10]. NT has been successfully applied to extract various volatile organic pollutants from air, water, exhalation [11], and blood samples [12]. NT has shown good performance in the extraction of airborne polycyclic aromatic hydrocarbons from diesel engine exhaust and pharmaceutical aerosols [13], which indicates the possibility to analyze volatile compounds in *Jinhua ham*.

SAFE is the conventional method with some limitations, SPME is frequently used in volatile compounds extraction for its simplicity and rapidity, and NT is an advanced technique used for food flavor detection. Therefore, in this study, SAFE, SPME, and NT were used to extract the aroma profile of *Jinhua ham*, and the extraction was further analyzed using gas chromatography–time-of-flight mass spectrometry (GC–TOF/MS). The objectives of this study were: (1) to find out the best extraction condition and compare the efficiencies of SAFE, SPME, and NT in extracting volatile aroma components of *Jinhua hams* at different aging times (one, two, and three years); (2) to characterize and identify the main volatile compounds in *Jinhua ham* using time-of-flight mass spectrometry; and (3) to explore the mechanisms of aroma profile formation of *Jinhua ham*. The results provide a better understanding of the aroma characteristics of *Jinhua ham*, and the conclusion can be used as a reference to extract flavor compounds in other muscle products.

## 2. Materials and Methods

### 2.1. Materials and Chemicals

**Figure 1:**
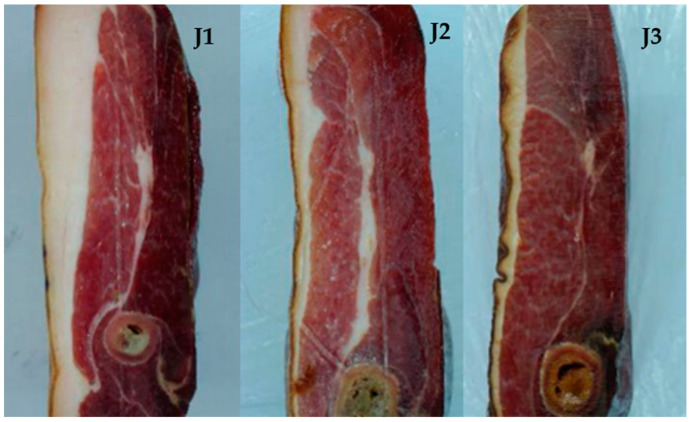
*Jinhua ham* samples aged for one (**J1**), two (**J2**), and three years (**J3**).

*Jinhua ham* samples were provided by Jinhua Jinnian Ham Co., Ltd. (Jinhua, Zhejiang, China). The aged times of the ham were one, two, and three years (see Figure 1). Different ham samples were sealed in vacuum bags and stored in a freezer at −80 °C until analysis.

All chemical standards used for identification and quantitation in this study were of chromatographic reagent grade unless otherwise stated: methanol (Mobe Biotechnology Co., Ltd., Shanghai, China); liquid nitrogen (Chlorine Gas Co., Ltd., Shanghai, China), and C_7_–C_40_ normal alkanes standard samples (Sigma Aldrich Trading Co., Ltd., Shanghai, China).

### 2.2. Instruments and Equipment

The ME204 electronic balance was from Mettler Toledo Instrument Co., Ltd. (Zurich, Switzerland); DF-101 collecting type thermostatic heating magnetic stirrer was from Yuhua Instrument Co., Ltd. (Gongyi, China); A11SSO025 analytical grinder was from Aika Instrument Equipment Co., Ltd. (Guangzhou, China); MD200-1 nitrogen blow dryer was from Aosheng Instrument Co., Ltd. (Hangzhou, China); 7890B GC was from Agilent and Pegasus BT TOF/MS from Leco (St. Joseph, MI, USA). The 50/30 μm DVB/CAR/PDMS SPME extraction head was from Shanghai Anpu Experimental Technology Co., Ltd. (Shanghai, China); 20 mL transparent headspace bottles were from Shanghai Anpu Scientific Instrument Co., Ltd. (Shanghai, China); a commercial needle trap with carboxen 1000 (internal adsorbent, 60–80 mesh) was from Shanghai Xintuo Analytical Instrument Technology Co., Ltd. (Shanghai, China).

### 2.3. Extraction of Volatile Compounds

#### 2.3.1. Sample Preparation

Sixty pig legs (10.0 ± 0.5 kg) were obtained from domestic pigs (Large White × Landrace; 6 months of age) of the same age in Jinhua and processed at Jinnian Ham Co., Ltd. (Jinhua, Zhejiang, China). The production of *Jinhua ham* involved salting, washing, and ripening of pig legs. The legs were rubbed with salt at 100 g/kg of weight in a curing room at temperature 3–5 °C and relative humidity of 80–89% for about a month. After salting, excess salt and impurities on the surface of ham needed to be washed away. The washed legs were transferred into the dry-ripening room. The temperature in the dry-ripening room was 25–37 °C and relative humidity was 60–70%. The hams were aged for one, two, and three years, respectively.

The ham stored in the −80 °C refrigerator was removed and thawed in a refrigerator at 4 °C overnight, then placed on a clean cutting board. Six legs were prepared for each aged *Jinhua ham*. After removing the leg bone and the epidermal fat layer, the remaining portion of ham (biceps femoris part) was cut into small pieces of 1 cm^3^.

#### 2.3.2. The Extraction of Aroma Compounds Using SAFE

The pretreated samples were ground into powder using an analytical grinder with the addition of liquid nitrogen. The powder sample (60 g) was weighed in a 500 mL sealed glass bottle, and 90 mL of dichloromethane was added thereto. After shaking for 12 h, the mixture was filtered using a separating funnel, and approximately 150 mL of organic phase was obtained. The extracts were evaporated again with SAFE (Kimble Bomex Labware Co., Ltd., Beijing, China) at 45 °C under high vacuum (7 × 10^−4^ Pa) [14]. Anhydrous Na_2_SO_4_ was added to remove water. The dichloromethane solution with the volatiles was slowly evaporated to 2 mL using a rotary evaporator, further concentrated to 0.5 mL under a nitrogen gas stream [5], then sealed and stored at −4 °C for GC–TOF/MS analysis within 12 h.

#### 2.3.3. The Extraction of Aroma Compounds Using SPME

We followed the reference to extract volatile compounds using the SPME method [15] and made some modifications. The grated ham samples (5 ± 0.01 g) were accurately weighed and placed in a 20 mL headspace flask, then the flask was sealed with PTFE-silicon stopper (Agilent). Partial cross-linked extraction fiber (CAR/PDMS) is appropriate for the extraction of trace volatile compounds, whereas the highly cross-linked extraction head (DVB/CAR/PDMS) is suitable for extracting the volatile compounds of C3–C20. In sum, the DVB/CAR/PDMS extraction fiber has the strongest adsorption capacity and was used in this study [16]. Before using the DVB/CAR/PDMS extraction head, it was activated at 250 °C for 30 min, and volatile compounds were extracted from samples at 60 °C for 40 min. Before the extraction, the vial was placed under the condition of a 60 °C constant temperature water bath for 10 min. Subsequently, the volatile compounds were extracted at 60 °C for 40 min.

#### 2.3.4. The Extraction of Aroma Compounds Using NT

A quantity of 5 ± 0.01 g of grated ham samples was accurately weighed and placed in a 20 mL headspace flask, then the flask was sealed with PTFE-silicon stopper (Agilent) before use. The air flow rate during sampling is one of the important parameters to be selected in needle trap technology. Appropriate sampling flow rate can minimize the whole sampling time without affecting the extraction efficiency. Before sampling, the needle trap was placed in the GC inlet at 250 °C for 15 min to remove pollutants [14]. A headspace bottle containing 5 g grated biceps femoris of *Jinhua ham* sample was placed on a headspace heater at 60 °C, and then a capture needle was connected with the portable sampler (flow rate: 2 mL/min). The needle trap was inserted into the headspace bottle containing the sample, and the needle tail was connected to the sampling box. When the air flow passed through the needle trap, the analyte was absorbed by the adsorbent, which is conducive to sampling. During analysis, the needle trap directly desorbed the analyte at the gas chromatography sample inlet. After enrichment for 120 min, the capture needle was detached and injected into the GC sample inlet (desorption temperature: 250 °C, desorption time: 5 min).

### 2.4. GC–TOF/MS Analysis

Aroma compounds in *Jinhua ham* extracted using the three methods were separated and identified using GC–TOF/MS equipped with a DB wax column (30 m × 0.25 mm, 0.25 μm, Agilent Technologies, Santa Clara, CA, USA). The GC conditions were as follows: the injector temperature was 250 °C, and helium (99.9999% purity) was used as the carrier gas at a constant flow rate of 1.0 mL/min in splitless injection mode. The temperature program of SPME and NT used was as follows: the oven temperature was kept at 40 °C for 4 min, then increased to 250 °C at a rate of 5 °C/min and held for 1 min. The temperature program of SAFE used was as follows: the oven temperature was kept at 40 °C for 3 min, then increased to 200 °C at a rate of 5 °C/min. The temperature was then increased to 250 °C at a rate of 10 °C/min, and finally held at 250 °C for 3 min.

The MS conditions were as follows: the temperature of the ion source was set at 220 °C, MS fragmentation was detected in electron-impact mode (ionization energy of 70 eV) with an acquisition range from 33 to 550 *m*/*z* in full-scan mode. The retention times of *n*-alkanes C_7_–C_40_ were used to calculate the retention indexes (RIs) of the volatile compounds in the ham samples.

### 2.5. Identification of Volatile Compounds

Under the aforementioned conditions of chromatography and mass spectrometry, the NIST 11.0 mass spectrometry library comparison method and RI method were used for qualitative analysis. The RI of each volatile component was calculated according to the *n*-alkane, matched with the NIST 11.0 database and compared with the RI value reported in the literature, then the target analyte was qualitatively determined.

$$\mathrm{RI}=\left(\frac{\mathrm{Rt}\left(x\right)-\mathrm{Rt}\left(n\right)}{\mathrm{Rt}\left(n+1\right)-\mathrm{Rt}\left(n\right)}\right)\times 100$$

where RI: retention index of volatile compounds to be tested; Rt(*x*): retention time of volatile compounds to be tested; Rt(*n*): retention time of *n*-alkanes with *n* carbon atoms; and Rt(*n* + 1): retention time of *n*-alkanes with *n* + 1 carbon atoms.

According to the peak areas of the compounds, the relative content of the volatile aroma components was determined using the area normalization method (ratio of peak area of individual volatile compound to total peak area).

### 2.6. Statistical Analysis

All of the data were analyzed using the GLM procedure of SAS software (version 8.0, SAS Institute, Cary, NC, USA). The fix effect of the treatments and the random effect of the replications were accounted for in the model. All the analyses were carried out in three replications. The differences in the mean values were accessed using Duncan’s multiple comparison method (*p* < 0.05). The principal component analysis (PCA) was performed using XLSTAT software (2020 version, Addinsoft, Redmond, WA, USA).

## 3. Results and Discussion

### 3.1. Volatile Compounds of Jinhua ham Extracted by SAFE, SPME, and NT

**Figure 2:**
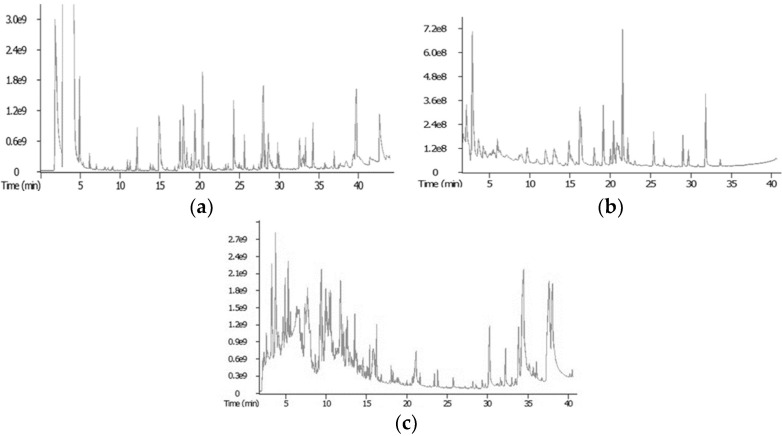
Total ionic chromatogram of volatile components in the three-year aged *Jinhua ham* extracted by SAFE (**a**), SPME (**b**), and NT (**c**).

SPME, NT, and SAFE methods were used to extract volatile compounds from *Jinhua ham* samples with different years of aging. In order to compare the extraction efficiency of the three methods, the extraction conditions were optimized, and we finally selected 60 °C as the best extraction temperature for the NT and SPME methods and 45 °C as the best temperature for the SAFE extraction method. Temperature has a great influence on the extraction effort of volatile compounds. If the extraction temperature is too high, it will destroy the original volatile compounds of the ham and produce some extra substances, and if the temperature is too low, volatiles in the sample cannot be volatilized. Therefore, a suitable extraction temperature is necessary. The total ion chromatograms by GC–TOF/MS are shown in Figure 2 (*Jinhua ham* aged for three years as an example). A total of 353 volatile compounds were identified as shown in Table S1 by the three extraction methods. Among the 353 volatile compounds, 134 volatiles were detected using SPME–GC–TOF/MS, and 205 volatiles were identified using NT–GC–TOF/MS, whereas 181 volatile compounds were found in *Jinhua ham* analyzed using SAFE–GC–TOF/MS. Table S1 shows that NT extracted the most types of compounds, followed by SAFE and SPME. This may be due to the dynamic needle of the NT device being filled with carboxen 1000, which had micropore and sub-micropore structures, so the extraction selectivity was higher. In addition, coupled with the continuous operation of the sampling pump, the enrichment effect was also higher. The volatile compounds identified using the three methods included aldehydes, ketones, alcohols, acids, esters, alkanes, alkenes, pyrazine, furan, and sulfides. In *Jinhua ham*, aldehydes were the dominant class of volatile compounds and major flavor contributors because of their low thresholds, which was consistent with previous studies [16,17].

The SPME method extracted 19 aldehydes in *Jinhua ham* samples, the NT method extracted 34 aldehydes, and the SAFE method extracted 29 aldehydes. Lipids in ham raw materials were hydrolyzed to form free fatty acids, then saturated and unsaturated fatty acids were converted to hydrogen peroxide and further reacted to form aldehydes [18]. Hexanal, octanal, nonanal, and benzaldehyde were identified in all of the *Jinhua ham* samples regardless of the age and extraction methods. As shown in Table S1, taking the *Jinhua ham* sample aged for one year as an example, the relative content of hexanal, octanal, nonanal, and benzaldehyde detected by SPME was significantly higher than that of NT and SAFE (*p* < 0.05). It may be because NT and SAFE detected more compounds, which led to the smaller relative content of individual substances. In addition, because the solvent peak can cover up the target aroma components, SAFE had limitations in the accurate quantification of volatile compounds. A previous study showed that hexanal came from the degradation of oxidized linoleic acid. To a certain extent, hexanal can be regarded as a measure of lipid peroxidation [19]. Because of the low aroma threshold, hexanal provided a pleasant grassy aroma to *Jinhua ham* [20]. Octanal and nonanal, which were derived from the oxidation of *n*–3 unsaturated fatty acids, gave *Jinhua ham* a meaty aroma [21]. Benzaldehyde, which had a bitter almond smell, was derived from reactions between reducing sugars and amino acids [22]. In addition to the aldehydes noted above, *trans*-2-octenaldehyde, phenylacetaldehyde, 3-methylbutyraldehyde, acetaldehyde, *cis*-2-heptanal, *trans*-2-nonenal, and *trans*-2-decenal were all detected using the three methods. Methyl-branched aldehydes such as 3-methylbutyraldehyde, which had the highest relevant percentage content detected using SPME (5.64–13.29%), originated from the degradation of leucine and contributed to the overall aroma of *Jinhua ham* by offering cheese, nutty, and salty notes that were associated with the cured and fermented aroma. Previous studies showed that 3-methylbutyraldehyde was the major aroma of Serrano, Iberian, Bayonne, and Corsican dry-cured hams due to its low odor threshold [23]. Careri et al. [24] reported that 3-methylbutyraldehyde was a key contributor to the aged flavor of Parma hams. Therefore, 3-methylbutyraldehyde can be regarded as the major aroma compound in *Jinhua ham*.

A total of 39 ketones were identified in *Jinhua ham* samples using the three methods. Among them, the SPME method extracted 7 kinds of ketones, the NT method extracted 22 ketones, and the SAFE method extracted 18. This may be due to SPME having a certain limitation for analyzing volatile compounds from dry-cured ham, especially for those with large molecular weight [25]. Ketones were mainly generated from two pathways, i.e., lipid oxidation and microbiological metabolism [26]. Lipid oxidation, through autoxidation, β-keto acid decarboxylation, or the β-oxidation of saturated fatty acids, mainly produced methyl ketones, which were considered as the precursors responsible for the fatty aroma in the ripened meat [22]. Methyl ketones, such as 2-heptanone, 2-nonanone, and 2-octanone, were associated with the aroma of blue cheeses and had an intense odor. Previous studies showed that 2-heptanone was the dominant ketone in dry-cured ham, whereas butanone and 2,3-butanedione were the major ketones in dry-cured bacon [27,28]. Methyl ketones can also be formed by chemical reactions in the presence of a large number of microorganisms, although a high concentration of ketones was a symptom of bad quality of *Jinhua ham* [29]. As an important aroma contributor to *Jinhua ham*, ketones were commonly associated with fruity, creamy, and cooked flavor characteristics [30]. The most abundant ketone in *Jinhua ham* detected using SPME was acetone (5.64–6.37%), which was significantly higher than when using NT and SAFE (*p* < 0.05). Using the NT extraction method, acetone was also the most abundant ketone (1.68~5.64%), but ketones only made up a small content in the overall volatile compounds, and the content of most ketones was less than 0.10%. As the most abundant ketone, acetone was detected using all three methods in the three different aging-time samples. 1-hydroxy-2-acetone was the most abundant ketone (2.37~7.73%) concentrated by SAFE, which was not detected by SPME and detected with a small relative amount (0.70%) in three-year aged ham sample by NT. Using SPME and SAFE, the content of many of the detected ketones showed a decreasing trend with the increase of aging time, which indicated that some ketones were transformed into carboxylic acids and other volatile components during the ripening phase [31].

Fifty-two alcohols were identified in the three different-aged *Jinhua ham* samples using the three methods. Among these, the SPME method extracted 16 types of alcohols, and the NT and SAFE methods extracted 35 and 30 alcohols, respectively. The alcohols detected in *Jinhua ham* included branched and linear alcohols, which were a high proportion. Studies showed that linear alcohols and some secondary alcohols were usually generated by the oxidation of polyunsaturated fatty acids, whereas branched-chain alcohols were mostly derived from microbial metabolism [32,33]. As a type of important volatile component in meat products, alcohols usually presented herb-like and woody tastes [26]. Ethanol and 1-octen-3-ol were the most abundant alcohols detected in *Jinhua ham*, which was consistent with the results of previous studies [2]. As a typical straight-chain aliphatic alcohol, ethanol can be generated by oxidation of lipids [32]. Ethanol was reported to have a high correlation with amino acids exclusively with creatine [22]. 1-Octen-3-ol had low threshold values and contributed a strong mushroom-like/earthy aroma to *Jinhua ham*, and it was present in higher amounts in volatile compounds detected using the SPME method [22]. A previous study showed that the β-oxidation of linoleic acid was the main pathway to form 1-octen-3-ol [34]. As typical products of lipolysis and lipid oxidation, 1-octen-3-ol and ethanol presented a high percentage content detected by SPME; this may be due to SPME having no solvent peaks to mask these peaks. Therefore, the SPME method played an important role in determining the number of alcohol compounds in the *Jinhua ham*.

As shown in Table S1, a total of 43 acids were identified in *Jinhua ham* samples. SAFE, NT, and SPME methods extracted 25, 18, and 36 acids, respectively. Acids were also one of the major components contributing to the unique flavor of *Jinhua ham*. Acids were usually formed by oxidation of aldehydes and enzymatic lipolysis during ham ripening. Linear-chain aliphatic acids can be derived from lipid oxidation or hydrolysis of triglycerides and phospholipids [35]. Acetic acid, butyrate, octanoic acid, decanoic acid, and 2-methylpropionic acid were common compounds found using the three extraction methods at each age point. These compounds were also the dominant acids in *Jinhua ham* samples and contributed stale fat flavor to *Jinhua ham* [36]. The main components of carboxylic acids were those with one to six carbon atoms, such as acetic acid, which provided *Jinhua ham* with an acidic flavor [31]. Acetic acid was the most abundant compound detected using the NT method, in which the percentage content of the three different-aged samples were all over 19%. Compared with other two extraction methods, the high volatility and low boiling points compounds were low and even not detectable with the SAFE method. For example, the relative peak area of acetic acid (4.52–12.15%) from the SAFE method was much lower than that from the NT method (19.18–29.51%). Because the pretreatment of the SAFE method was carried out at a relatively lower temperature (45 °C) and under high-vacuum, the distillates contained fewer high boiling point volatiles. Also, the SAFE method was more effective in extracting fewer volatile and polar components. With the increase in aging time, the content of acetic acid increased. This may be due to the Maillard reactions [25]. In addition, the relative carboxylic acid content of *Jinhua ham* was higher due to its maturation temperature being about 5–8 °C higher than that of Mediterranean dry-cured ham. This was also a major factor contributing to the unique flavor of *Jinhua ham* [25].

Seventy-four esters were identified in the three selected aging hams using the three methods. Among these esters, SPME extracted 25 esters, NT extracted 37 esters, and SAFE extracted 35 esters. Esters strongly affected the overall aroma of *Jinhua ham* as a typical traditionally aged meat product. In particular, the methyl-branched short-chain esters were found to positively contribute to the flavor of aged meat [16]. Methyl esters, such as methyl acetate and methyl butyrate, can be formed by the esterification activities of staphylococci and lactic acid bacteria [37]. During the process of ham ripening, esters were formed by the esterification of carboxylic acids and alcohols in musculature. Because of their low odor thresholds, esters contribute to the overall aroma, hence conferring fruit and fat flavors to dry-cured ham. Esters with short-chain acids presented a fruity aroma, whereas those generated with long-chain acids have a fatty odor [38]. Among the esters, four of them were common volatiles detected using the three methods: ethyl decanoate, dehydropropionolactone, propyl acetate, and butyrolactone. Using SPME, propyl acetate was the most abundant ester, constituting 5.93% of the total volatiles in two-year-aged ham, which was significantly higher than from the other two methods (*p* < 0.05). Propyl acetate provided *Jinhua ham* with a soft fruity aroma because of its short-chain acid. Except for propyl acetate, methyl hexanoate (1.11~2.75%) and methyl isovalerate (1.53~2.59%) presented a higher percentage content detected by SPME. In general, most of esters detected in *Jinhua ham* were methyl and ethyl esters, which can be formed by the esterification of ethanol and carboxylic acids with the involvement of microorganisms [39]. Forty-six alkanes and twenty olefins were identified in *Jinhua ham* using the three methods. The production of alkanes was closely related to lipid oxidation and thermal decomposition, mainly from the cleavage of aliphatic acid alkoxy radicals [26]. Alkanes had higher odor thresholds, so they made little contribution to the overall flavor of *Jinhua ham*. SPME extracted the most types of hydrocarbons. Among the detected alkanes using the SPME method, decane was the most abundant compound (1.57~4.25%), which was significantly higher than the levels detected by NT and SPME (*p* < 0.05). In addition, hydrocarbons with longer chains, such as dodecane and tridecane, also had a higher content, which might be from feeding. Hydrocarbons with less than 10 carbon atoms were mainly generated from lipid oxidation [40]. It was worth noting that using the SAFE method, the relative percentage contents of *n*-hexane were 34.46%, 32.53%, and 16.18% of one-, two-, and three-years-aged samples.

In addition to the volatile compounds noted above, pyrazine, furan, and sulfides were also detected in *Jinhua ham* using the three extraction methods, and they played an important role in the overall flavor of *Jinhua ham*. The nutty and baking flavors of dry-cured ham were provided by pyrrole and 2,6-dimethylpyrazine [41], whereas sulfur compounds, such as dimethyl disulfide and dimethyl trisulfide, which formed through the reaction of sulfur-containing amino acids with carboxylic compounds, were the key aroma compounds of dry-cured ham [14]. The sulfides, including dimethyl sulfide, dimethyl disulfide, dimethyl trisulfide, and dimethyl tetrasulfide, mainly came from Maillard reactions and thiamine degradation, with garlic flavor and a strong smell of cooked ham [42]. The aroma of dry-cured ham can also be formed through microbial metabolism [43]. Pyrazine and pyrrole were the main products of microbial degradation [44] and were the key volatile aroma compounds with baking and nut smells [43]. The degradation of thiamine produces volatile aroma components, such as furan, and the amount of these secondary degradation products depends on the heating temperature, time, pH, and matrix compositions [45]. These compounds had very low odor thresholds, which was very important to the overall flavor of meat products, and contributed to the production of grass flavor, meat flavor, and baking flavor [46]. In addition, phenols, such as cresols, phenol, 3-methoxyphenol, and *p*-cresol, can provide a smoky flavor for ham [47].

### 3.2. Comparison Analysis of Three Pretreatment Methods

The results of SAFE, SPME, and NT extraction methods were different, and the differences were presented in two aspects: the amounts and relative percentage content of the extracted volatile compounds. The three methods were mainly divided into headspace extraction and solvent extraction. In the two headspace methods (SPME and NT), SPME mainly relies on the fiber coating material of the extraction head. SPME uses quartz fiber heads with different polar layers to statically adsorb volatile compounds in the headspace of the sample flask. Different fiber coating materials of SPME have different extraction effect of volatile compounds, such as non-bonded cross-linked extraction fiber (PDMS), which is more suitable for extracting small molecule compounds, nonpolar compounds, and volatile and semi-volatile compounds. In this study, a total of 134 volatile compounds were detected by SPME.

NT is a solvent-free extraction method. When the air in the headspace of the sample is driven through the adsorption bed of the needle trap, the volatile compounds are adsorbed by the adsorbent in the adsorption bed. Different filler materials of the NT device have different abilities to adsorb volatile compounds from a complex matrix. The Carboxen 1000 extraction needle used in this study has good adsorption and desorption performance, the special small cross-section geometry of the extraction needle does not need to be calibrated, and the structure of the device has good storage stability [10]. A total of 205 volatile compounds were detected by NT, which was more than by SPME and SAFE. Compared with SPME, the NT device has a constant-current pump that constantly blows the air in the sample bottle into the needle trap, which is a dynamic headspace extraction technology. In addition, the whole enrichment process of NT lasted for 120 min, which was longer than for SPME (40 min). Therefore, NT method can extract more volatile compounds than SPME.

As the pretreatment of the SAFE method was carried out under a high vacuum (7 × 10^−4^ Pa) and relative lower temperature (45 °C), the distillate contained fewer high boiling point volatile compounds. A total of 181 volatile compounds were detected by SAFE. In terms of the types of compounds extracted, the SAFE method was slightly higher than SPME, indicating that the solvent extraction rate was higher than with the headspace method. However, due to the large solvent peak, which covers the target aroma components, SAFE has some limitations in accurate quantification [5].

**Figure 3:**
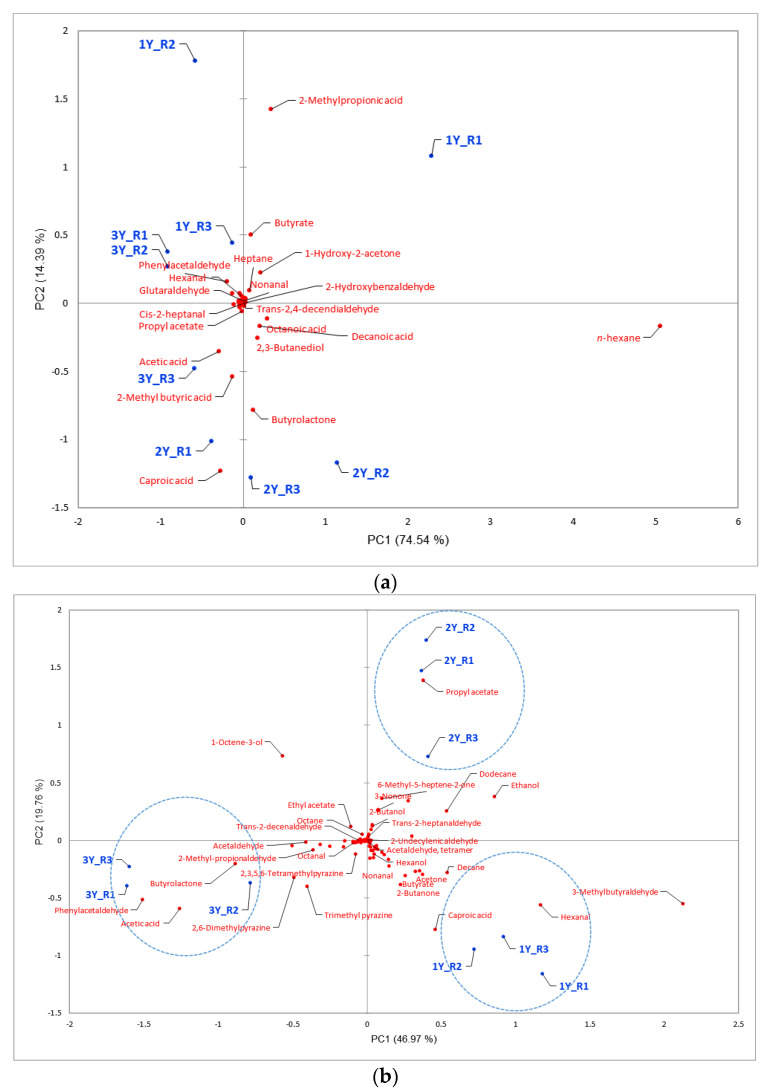

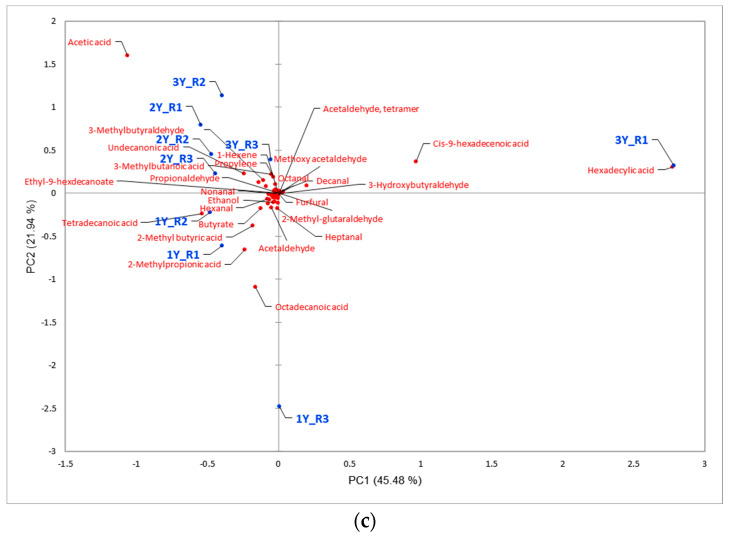
Principal component analysis of volatile components of *Jinhua hams* at different points in the aging process (1Y, 2Y, and 3Y) using SAFE (**a**), SPME (**b**), and NT (**c**); R1, R2, R3 represent three replications of each aged ham.

To establish the relationship between extraction methods and detected volatile composition, a PCA was applied. As shown in Figure 3b, the first principal component, PC1, accounted for 46.97% of the sample variance, and the second, PC2, for 19.76%. The lower-right quadrant of the principal component plot indicated that the volatile profile of *Jinhua ham* that had aged for one year was associated with caproic acid, hexanal, and 3-methylbutyraldehyde, which provided *Jinhua ham* with stale fat, grass, and fruit aromas, respectively [2,36]. The upper-right quadrant showed that propyl acetate was the main volatile compound of two-year-aged ham samples, which presented a mild fruity aroma [38]. Butyrolactone (milk and cream odor), phenylacetaldehyde (acorn, rancid, spicy odor) [36], 2,3,5-trimethylpyrazine (chocolate, peanuts odor), 2,6-dimethylpyrazine (roast, coffee odor) [43], and acetic acid (acidic odor) [31] were associated more with three-year-aged ham samples. From the statistical point of view, these compounds were regarded as the major volatile compounds of three-year-aged ham. Therefore, it can be seen that PCA of SPME could effectively distinguish the variation in the aroma of the *Jinhua hams* specific to aging time.

As Figure 3a shows, the first and second principal components accounted for 74.54% and 14.39% of the sample variance, respectively. By the extraction method of SAFE, 2-methylpropionic acid and butyrate were considered as the main aroma compounds in one-year-aged samples, caproic acid and butyrolactone were the main compounds in two-year-aged samples. As for three-year-aged ham samples, acetic acid and phenylacetaldehyde were considered as the main volatile compound contributors, which agrees with the results of SPME. Figure 3c shows the PCA of flavor compounds of *Jinhua hams* at different points in the aging process by NT. The first principal component (PC1) explained 45.48% and the second principal component (PC2) explained 21.94% of the variations. It can be seen from the figure that tetradecanoic acid, 2-methylbutyric acid, 2-methylpropionic acid, and octadecanoic acid were the major components in one-year-aged samples, whereas for the two- and three-year-aged samples, the main volatile compounds were acetic acid and undecanoic acid. Therefore, NT was a better method for the extraction of acids. As we discussed previously, acids were usually generated by neutral fat degradation, amino acid deamination, or microbiological metabolism, and they provide *Jinhua ham* with stale fat, cheesy, or acidic odors [2]. Generally speaking, the ability of the NT and SAFE methods to distinguish the variation of three different-aged *Jinhua hams* was not as good as SPME.

## 4. Conclusions

In this study, the SAFE, SPME, and NT methods were used to extract the volatile aroma compounds of *Jinhua ham*. GC–TOF/MS was used to identify the volatile aroma compounds of *Jinhua ham* in different-aged years. A total of 355 volatile compounds were identified in *Jinhua ham*, and the major volatile compounds were aldehydes, ketones, alcohols, and acids. The results showed that NT extracted the greatest number of volatiles and was especially more efficient in the concentration of acid compounds. The SPME method played an important role in determining alcohol compounds because it has no solvent peaks to mask these peaks. The use of PCA to assess the variation of volatiles extracted using the three pretreatment methods indicated that SPME was more effective in distinguishing the variation in the aroma of the *Jinhua hams* specific to aging time. In conclusion, SPME was the most effective method in the volatile compounds detection, and the results of SAFE, SPME, and NT were complementary. This study can provide a theoretical basis for the selection of *Jinhua ham* volatile aroma compounds extraction methods.

## Data Availability

The data used to support the findings of this study can be made available by the corresponding author upon request.

## Supplementary Materials

The following supporting information can be downloaded at: https://www.mdpi.com/article/10.3390/foods11233897/s1, Table S1: Identified volatile components in *Jinhua ham* by three extraction methods (SPME, NT and SAFE).
