# Supplementary material for: Analysis of Volatile Compounds in Jinhua ham Using Three Extraction Methods Combined with Gas Chromatography–Time-of-Flight Mass Spectrometry
Source: Foods. 2022 Dec 2;11(23):3897. doi: 10.3390/foods11233897 (PMC9735730; doi:10.3390/foods11233897)
Supplement: Supplementary file 1 [file foods-11-03897-s001.zip › foods-2004329-supplementary.pdf]

Table S1. Identified volatile components in *Jinhua ham* by three extraction methods (SPME, NT and SAFE)

Compounds [1]	RI [2]	CAS	Identification method	Relative percentage content (%)								
				SPME			NT			SAFE		
				**One- year aging**	**Two- year aging**	**Three- year aging**	**One- year aging**	**Two- year aging**	**Three- year aging**	**One- year aging**	**Two- year aging**	**Three- year aging**
				Aldehyde								
Hexanal	1069.1	66-25-1	RI, MS	5.69±2.26^{a}	3.57±1.83^{bc}	0.84±0.11^{c}	1.45±1.37^{bc}	2.02±1.35^{bc}	0.48±0.44^{c}	1.21±0.91^{bc}	0.54±0.15^{c}	0.69±0.21^{c}
Octanal	1353.7	124-13-0	RI, MS	1.15±1.32^{ab}	1.08±1.08^{ab}	1.53±0.59^{a}	0.08±0.08^{b}	0.00±0.01^{b}	0.28±0.04^{b}	0.14±0.09^{b}	0.05±0.01^{b}	0.10±0.04^{b}
Nonanal	1483.2	124-19-6	RI, MS	2.73±1.37^{a}	1.88±0.25^{a}	1.97±0.47^{a}	0.43±0.30^{b}	0.66±0.41^{b}	0.28±0.04^{b}	0.49±0.24^{b}	0.16±0.03^{b}	0.36±0.11^{b}
Acetaldehyde	531.1	75-07-0	RI, MS	0.35±0.30	0.69±0.63	2.15±0.33	2.50±2.50	-	2.49±1.87	-	-	-
Trans-2-octenaldehyde	1509.4	2548-87-0	RI, MS	0.07±0.04^{b}	0.11±0.03^{b}	0.23±0.04^{a}	0.20±0.08^{a}	0.06±0.04^{b}	0.05±0.02^{b}	0.05±0.03^{b}	0.02±0.01^{b}	0.05±0.02^{b}
2-Methyl-propionaldehyde	595.2	78-84-2	RI, MS	1.21±1.05^{b}	1.60±0.16^{b}	2.97±0.21^{a}	0.89±0.70^{b}	0.68±0.95^{b}	0.29±0.24^{b}	-	-	-
3-Methylbutyraldehyde	740.6	590-86-3	RI, MS	13.29±0.57^{a}	11.50±2.20^{a}	5.64±2.68^{b}	0.36±0.26^{c}	2.48±3.17^{bc}	1.58±1.12^{bc}	-	-	0.08±0.08^{c}
Benzaldehyde	1553.3	100-52-7	RI, MS	2.17±0.31^{a}	1.82±0.07^{a}	2.04±0.16^{a}	0.49±0.23^{b}	0.69±0.20^{b}	0.47±0.18^{b}	0.58±0.29^{b}	0.28±0.05^{b}	0.34±0.17^{b}
Acetaldehyde, tetramer	2483	108-62-3	RI	0.05±0.05	0.01±0.00	0.01±0.01	0.19±0.27	0.03±0.04	0.01±0.01	-	-	-
Phenylacetaldehyde	1595.6	122-78-1	RI, MS	5.04±0.43^{b}	5.21±0.63^{b}	11.76±2.16^{a}	-	-	0.47±0.33^{c}	4.35±3.87^{bc}	1.39±1.22^{bc}	3.16±3.36^{bc}
Trans-2-decenaldehyde	1630.8	3913-81-3	RI, MS	0.06±0.05^{b}	0.12±0.08^{ab}	0.30±0.09^{a}	-	-	0.19±0.16^{ab}	0.06±0.08^{b}	0.09±0.12^{ab}	-
Propionaldehyde	578.1	123-38-6	RI, MS	0.02±0.03	-	-	-	-	0.00±0.00	-	-	-
Furfural	1514.8	1998-1-1	RI, MS	-	-	0.11±0.16	0.07±0.05	-	0.06±0.04	-	-	0.00±0.00
Methoxy acetaldehyde	2909.8	10312-83-1	RI	0.01±0.01	-	-	-	-	0.22±0.16	-	-	-
Trans-2-heptanaldehyde	1412.9	18829-55-5	RI, MS	-	0.12±0.12	-	-	-	-	-	-	-
2-Undecylenic aldehyde	1759.8	2463-77-6	RI, MS	-	0.02±0.02	-	-	-	-	-	-	-
5-Hexenal	1879	764-59-0	RI	-	-	0.01±0.00	-	-	-	-	-	-

Cis-2-heptanal	1380.7	57266-86-1	RI, MS	-	-	0.97±0.40[a]	0.17±0.13[b]	-	-	-	0.06±0.01[b]	0.29±0.16[b]
Trans-2-nonenal	1556.1	18829-56-6	RI, MS	-	-	0.13±0.11	-	-	0.04±0.05	-	-	0.03±0.01
Decanal	1530.7	112-31-2	RI, MS	-	-	-	0.03±0.03[b]	0.06±0.05[b]	0.33±0.19[a]	0.06±0.06[b]	0.01±0.01[b]	-
Heptanal	1161	111-71-7	RI, MS	-	-	-	1.30±1.71	0.30±0.39	-	0.11±0.06	0.05±0.01	0.09±0.03
2,4-Decendialdehyde	1765.2	2363-88-4	RI, MS	-	-	-	0.13±0.15	-	0.20±0.12	0.14±0.06	-	-
Trans-2-undecylenic aldehyde	1716.7	2463-77-6	RI, MS	-	-	-	0.07±0.07	-	0.09±0.05	-	-	0.01±0.01
3-Hydroxybutyraldehyde	1156.5	107-89-1	RI	-	-	-	0.03±0.05	-	-	-	-	-
2-Methyl-glutaraldehyde	1266.3	123-15-9	RI	-	-	-	0.00±0.00	-	-	-	-	-
4-Ethylbenzaldehyde	1699	4748-78-1	RI, MS	-	-	-	0.03±0.01	-	-	-	-	-
Trans-2,4-decendialdehyde	1716.9	25152-84-5	RI, MS	-	-	-	0.04±0.04	-	-	0.07±0.04	0.06±0.02	0.05±0.05
2,3-Dimethyl-glutaraldehyde	820.8	32749-94-3	RI, MS	-	-	-	-	0.57±0.81	-	-	-	-
2-Butyraldehyde	1058.4	1119-19-3	RI	-	-	-	-	0.00±0.00	-	-	-	0.02±0.02
3,4-Glutaraldehyde	1063.6	4009-55-6	RI	-	-	-	-	0.00±0.00	-	-	-	-
2-Nonenal	1554.6	2463-53-8	RI, MS	-	-	-	-	0.06±0.05	-	0.03±0.01	0.01±0.01	-
2-Methyl-2-butyraldehyde	1317.5	1115-11-3	RI, MS	-	-	-	-	-	0.00±0.00	0.02±0.01	-	-
5-Methyl furfural	1638.3	620-02-0	RI, MS	-	-	-	-	-	0.05±0.06	-	-	-
Undecylaldehyde	1662.7	112-44-7	RI, MS	-	-	-	-	-	0.05±0.06	-	-	-
3-Methyl-benzaldehyde	1679.4	620-23-5	RI, MS	-	-	-	-	-	0.01±0.01	-	-	-
Methylal	2060.6	109-87-5	RI	-	-	-	-	-	0.04±0.05	-	-	-
5-Hydroxymethylfurfural	2544.1	67-47-0	RI	-	-	-	-	-	0.23±0.09	-	-	-
Glutaraldehyde	1004.2	110-62-3	RI, MS	-	-	-	-	-	-	0.48±0.27	0.28±0.09	0.37±0.13
2-Hexenal	1184.6	505-57-7	RI, MS	-	-	-	-	-	-	0.00±0.00	0.00±0.00	0.00±0.00
3-Methyl-2-butyraldehyde	1167.4	107-86-8	RI, MS	-	-	-	-	-	-	0.03±0.02	0.02±0.01	0.04±0.03

2-Hydroxybenzaldehyde	1605.7	1990-2-8	RI, MS	-	-	-	-	-	-	0.04±0.02	0.02±0.01	-
2,6-Dimethyl-5-heptanal	1030.7	106-72-9	RI, MS	-	-	-	-	-	-	0.01±0.01	-	0.00±0.00
2-Methyl-2-pentenal	1056.7	623-36-9	RI, MS	-	-	-	-	-	-	0.00±0.00	-	-
Tetradecane aldehyde	1847.4	124-25-4	RI, MS	-	-	-	-	-	-	0.00±0.00	-	-
4-Methoxybenzaldehyde	1940.3	123-11-5	RI, MS	-	-	-	-	-	-	-	0.01±0.01	-
Trans-2,6-nondialdehyde	1258	17587-33-6	RI, MS	-	-	-	-	-	-	-	-	0.00±0.00
Trans-2,4-heptadienaldehyde	1517	4313-3-5	RI, MS	-	-	-	-	-	-	-	-	0.01±0.01
						Ketones						
Acetone	600.2	67-64-1	RI, MS	6.37±5.55^{a}	6.11±0.33^{ab}	5.64±2.68^{ab}	1.68±0.82^{c}	2.19±1.50^{bc}	1.07±0.12^{c}	0.91±0.30^{c}	0.78±0.25^{c}	0.49±0.47^{c}
2-Butanone	710.8	78-93-3	RI, MS	4.09±0.56^{a}	2.39±0.54^{b}	2.62±0.44^{b}	0.76±0.39^{c}	-	1.38±0.97^{bc}	-	-	-
3-Undecanone	1578.1	2216-87-7	RI	0.01±0.01	0.02±0.00	-	-	-	-	-	-	-
3-Nonone	1478.2	925-78-0	RI, MS	-	1.22±0.20	-	-	-	-	-	-	-
2,3-Octadione	1416.8	585-25-1	RI, MS	-	1.22±0.20^{a}	-	-	-	0.06±0.06^{b}	-	0.06±0.02^{b}	-
2,3-Pentanedione	1552.4	600-14-6	RI, MS	-	-	0.00±0.00^{b}	-	-	-	0.02±0.02^{ab}	0.02±0.01^{ab}	0.04±0.02^{a}
6-Methyl-5-heptene-2-one	1425.4	110-93-0	RI, MS	-	1.57±0.43	-	-	-	-	-	-	-
2-Pentadecanone	1990.5	2345-28-0	RI, MS	-	-	-	0.23±0.03	0.17±0.03	-	-	-	-
2-Heptanenone	2194.7	2922-51-2	RI, MS	-	-	-	0.04±0.03	0.07±0.04	-	-	-	-
2-Octanone	1518.9	111-13-7	RI, MS	-	-	-	0.04±0.01	-	0.02±0.03	-	-	-
3-Pentanone	873.2	96-22-0	RI, MS	-	-	-	0.30±0.40	-	-	-	-	-
2,5-Hexanedione	1359.3	110-13-4	RI, MS	-	-	-	0.04±0.04	-	-	-	-	-
Amyl ketone	1968.9	942-92-7	RI	-	-	-	0.06±0.00	-	-	-	-	-
4-Methyl-2-heptanone	620.6	6137-06-0	RI, MS	-	-	-	-	0.01±0.02	-	-	-	-
2,3-Butanedione	894.4	431-03-8	RI, MS	-	-	-	-	0.16±0.22	-	-	-	-

1-Pentene-3-one	1172.9	1629-58-9	RI, MS	-	-	-	-	0.03±0.04	-	-	-	-
P-ethyl Acetophenone	1792.7	937-30-4	RI, MS	-	-	-	-	0.09±0.02	-	-	-	-
4-(4-Hydroxy-3-methoxyphenyl)-2-butanone	2644.1	122-48-5	RI, MS	-	-	-	-	0.16±0.13	-	-	-	-
2-Methyl-4-octanone	1410.8	7492-38-8	RI	-	-	-	-	-	0.00±0.00	-	-	-
1-Hydroxy-2-acetone	1456	116-09-6	RI, MS	-	-	-	-	-	0.70±0.32^{c}	7.73±2.79^{a}	3.93±1.11^{b}	2.37±1.17^{bc}
2-Heptanone	1527	110-43-0	RI, MS	-	-	-	-	-	0.06±0.06^{b}	0.09±0.06^{a}	0.10±0.01^{a}	0.00±0.00^{b}
2,3-Hexanedione	1918.6	3848-24-6	RI, MS	-	-	-	-	-	0.07±0.10	-	-	-
5-Methyl-1-phenyl-1-hexone	2090.2	25552-17-4	RI	-	-	-	-	-	0.08±0.03	-	-	-
Dodecanone	2104.8	6175-49-1	RI, MS	-	-	-	-	-	0.01±0.01	-	-	-
7-Octene-2-one	2119.8	3664-60-6	RI	-	-	-	-	-	0.03±0.02	-	-	-
2-Tetradecanone	2311.8	2345-27-9	RI	-	-	-	-	-	0.01±0.01	-	-	-
3-Octanone	1236.4	106-68-3	RI, MS	-	-	-	-	-	-	0.01±0.01	0.01±0.00	0.01±0.01
3-Methyl-2-butanone	908.9	563-80-4	RI, MS	-	-	-	-	-	-	0.10±0.10	0.33±0.17	0.16±0.05
4-Hydroxy-4-methyl-2-pentanone	1412.3	123-42-2	RI, MS	-	-	-	-	-	-	0.06±0.03	0.04±0.01	0.04±0.02
2-Hexone	1012.4	591-78-6	RI, MS	-	-	-	-	-	-	0.07±0.03	0.05±0.01	-
2-Tridecanone	1739.5	593-08-8	RI, MS	-	-	-	-	-	-	0.09±0.04	0.06±0.02	-
1-Hydroxy-2-butanone	1420.4	5077-67-8	RI, MS	-	-	-	-	-	-	0.01±0.01	-	0.09±0.04
4-Methyl-2-hexone	1106.4	105-42-0	RI	-	-	-	-	-	-	0.01±0.01	-	-
2-Nonanone	1439.5	821-55-6	RI, MS	-	-	-	-	-	-	-	-	0.02±0.02
1-Octene-3-one	1317.6	4312-99-6	RI, MS	-	-	-	-	-	-	-	-	0.08±0.03
3-Pentene-2-one	1010.5	625-33-2	RI, MS	-	-	-	-	-	-	-	-	0.46±0.37
2-Methyl-3-heptanone	1144.3	13019-20-0	RI	-	-	-	-	-	-	-	-	0.06±0.01
1-Methoxy-2-acetone	1087.1	5878-19-3	RI, MS	-	-	-	-	-	-	-	-	0.00±0.00

4,6-Dimethyl-2-heptanone	1106.4	19549-80-5	RI	-	-	-	-	-	-	-	-	0.00±0.01
Alcohols												
N-octanol	1574.3	111-87-5	RI, MS	0.45±0.24^{a}	0.55±0.24^{a}	0.64±0.09^{a}	0.04±0.74^{b}	-	-	0.33±0.31^{ab}	-	-
Benzyl alcohol	1849.9	100-51-6	RI, MS	0.06±0.05^{d}	0.09±0.01^{d}	0.11±0.03^{cd}	-	0.04±0.03^{d}	0.04±0.01^{d}	0.22±0.08^{ab}	0.19±0.04^{bc}	0.31±0.09^{a}
Phenylethanol	1884.4	1960-12-8	RI, MS	0.31±0.10^{c}	0.59±0.42^{bc}	0.95±0.43^{bc}	-	0.08±0.02^{c}	0.03±0.02^{c}	1.87±1.56^{ab}	0.95±0.29^{bc}	2.99±1.04^{a}
2-Ethyl-1-hexanol	1541.1	104-76-7	RI, MS	0.08±0.01^{a}	0.04±0.04^{b}	0.03±0.01^{b}	-	-	-	0.03±0.02^{b}	0.02±0.02^{b}	0.02±0.01^{b}
2-Ethyl-1-decanol	1845.4	21078-65-9	RI, MS	0.02±0.02	0.02±0.01	0.02±0.01	-	-	-	-	-	-
Furfuryl alcohol	1668.4	98-00-0	RI, MS	-	0.05±0.04	0.04±0.01	0.13±0.15	0.24±0.07	-	-	-	-
Ethanol	781.2	64-17-5	RI, MS	3.34±1.01	4.26±2.86	-	1.88±0.64	-	3.9±3.22	-	-	-
2-Butanol	2821.5	78-92-2	RI	0.03±0.04	0.54±0.86	-	-	0.13±0.19	-	0.00±0.00	0.03±0.01	0.09±0.05
Trans-2-octene-1-ol	1600.2	18409-17-1	RI, MS	0.85±0.69	2.27±0.03	-	-	-	-	-	-	-
Hexanol	1443.7	111-27-3	RI, MS	0.58±0.62	-	0.24±0.06	0.05±0.06	0.12±0.02	0.04±0.05	0.10±0.15	0.06±0.02	0.06±0.02
1-Octene-3-ol	1520.9	3391-86-4	RI, MS	-	4.47±0.60^{a}	3.67±0.11^{b}	-	0.23±0.05^{c}	0.39±0.48^{c}	0.61±0.49^{c}	0.37±0.06^{c}	0.59±0.18^{c}
3-Methylthiopropanol	1693.5	505-10-2	RI, MS	-	0.06±0.04	0.08±0.02	-	-	-	-	-	-
5-Nonanol	1742	623-93-8	RI	-	-	0.02±0.00	-	-	-	-	-	-
2-Methoxy-ethanol	786.7	109-86-4	RI, MS	-	-	0.02±0.02	-	-	0.01±0.01	-	-	-
2-Methyl-3-pentanol	1084.4	565-67-3	RI	-	-	0.29±0.26	-	-	-	0.07±0.04	-	0.02±0.02
1-Pentene-3-ol	1158.6	616-25-1	RI, MS	-	-	0.36±0.08^{a}	-	-	-	0.22±0.07^{b}	0.13±0.03^{b}	0.19±0.05^{b}
1-Dodecanol	1936.5	112-53-8	RI, MS	-	-	-	0.03±0.02	0.08±0.03	0.12±0.04	0.02±0.02	0.08±0.12	-
2,3-Butanediol	1577.3	513-85-9	RI, MS	-	-	-	1.69±0.58	1.21±0.87	0.04±0.04	-	2.42±2.68	-
N-pentanol	1328.4	71-41-0	RI, MS	-	-	-	0.09±0.13^{c}	0.41±0.05^{a}	0.26±0.24^{ab}	0.16±0.13^{ab}	0.06±0.02^{c}	0.09±0.03^{c}
3-Methyl-1-butanol	1190.3	123-51-3	RI, MS	-	-	-	-	0.32±0.29	0.41±0.58	0.25±0.20	0.10±0.06	0.19±0.06
Isopropanol	740.4	67-63-0	RI, MS	-	-	-	0.10±0.12	-	0.06±0.06	-	-	-

1-Butanol	556	71-36-3	RI, MS	-	-	-	0.24±0.33	-	0.16±0.23	0.11±0.08	0.03±0.04	0.07±0.04
1-Heptanol	1517.5	111-70-6	RI, MS	-	-	-	0.11±0.11	-	-	-	0.02±0.02	-
2,2'-Oxydiethanol	1931.3	111-46-6	RI, MS	-	-	-	0.05±0.03	-	-	-	-	-
1,4-Butanediol	784.9	110-63-4	RI	-	-	-	-	0.08±0.11	-	-	-	-
2-Ethyl-1-butanol	1029.3	97-95-0	RI, MS	-	-	-	-	0.02±0.02	-	-	-	-
1-Methoxy-2-propanol	1057.7	107-98-2	RI, MS	-	-	-	-	0.07±0.05	-	0.06±0.04	0.10±0.03	0.12±0.07
2-Methyl-1-propanol	1086.3	78-83-1	RI, MS	-	-	-	-	0.15±0.21	-	0.07±0.06	0.09±0.06	0.15±0.02
N-propanol	1094.6	71-23-8	RI, MS	-	-	-	-	-	0.02±0.02	-	-	-
3-Butene-2-ol	1292	598-32-3	RI	-	-	-	-	-	0.01±0.01	-	-	-
2-Ethoxy-ethanol	1423.2	110-80-5	RI, MS	-	-	-	-	-	0.01±0.01	-	-	-
2-Methyl-3-hexanol	1427.6	617-29-8	RI	-	-	-	-	-	0.03±0.04	-	-	-
4-Octanol	1524.3	589-62-8	RI	-	-	-	-	-	0.00±0.01	-	-	-
1-Nonanol	1733	143-08-8	RI, MS	-	-	-	-	-	0.02±0.02	-	-	-
2-Furan methanol	1733.4	98-00-0	RI, MS	-	-	-	-	-	0.06±0.06	-	-	-
3-(Methylthio)-1-propanol	1793.1	505-10-2	RI, MS	-	-	-	-	-	0.01±0.01	-	-	0.25±0.05
2-(2-Hydroxypropoxy)-1-propanol	1974.7	106-62-7	RI, MS	-	-	-	-	-	0.03±0.03	-	-	-
4-Heptanol	1978.3	589-55-9	RI, MS	-	-	-	-	-	0.03±0.02	-	-	-
2-Methylbenzyl alcohol	2075.6	89-95-2	RI	-	-	-	-	-	0.01±0.01	-	-	-
N-decanol	2259.4	112-30-1	RI, MS	-	-	-	-	-	0.01±0.01	-	-	-
Hexadecanol	2446.8	36653-82-4	RI, MS	-	-	-	-	-	0.08±0.03	-	0.10±0.14	-
2-Pentanol	2702.1	6032-29-7	RI, MS	-	-	-	-	-	0.02±0.02	-	-	0.06±0.06
3-Pentene-2-ol	1146.7	1569-50-2	RI, MS	-	-	-	-	-	-	0.12±0.06[a]	0.04±0.03[b]	0.02±0.02[b]
2,2'-Oxybis ethanol	1895.2	111-46-6	RI, MS	-	-	-	-	-	-	0.01±0.01	0.01±0.01	0.05±0.05

2-Methyl-3-butene-2-ol	1043.1	115-18-4	RI, MS	-	-	-	-	-	-	0.20±0.08	0.10±0.03	0.44±0.33
3-Methyl-3-butene-1-ol	1226	763-32-6	RI, MS	-	-	-	-	-	-	0.01±0.00	0.02±0.01	-
2-Methyl-2-propanol	1210.7	75-65-0	RI, MS	-	-	-	-	-	-	0.01±0.01	-	-
3-Pentanol	1535.9	584-02-1	RI, MS	-	-	-	-	-	-	0.00±0.01	-	-
Octadecyl alcohol	2614.2	112-92-5	RI, MS	-	-	-	-	-	-	0.01±0.02	-	-
Cis-3-hexenol	1430.4	928-96-1	RI, MS	-	-	-	-	-	-	-	0.02±0.03	-
3-Octanol	2469.9	589-98-0	RI, MS	-	-	-	-	-	-	-	-	0.15±0.06
2-Methyl-1-pentene-3-ol	2041.8	2088-7-5	RI, MS	-	-	-	-	-	-	-	-	2.45±0.85
							Acids					
Acetic acid	1518.2	64-19-7	RI, MS	4.85±1.29^{c}	4.02±0.99^{c}	9.33±2.90^{bc}	19.18±13.77^{abc}	29.51±4.20^{a}	25.08±11.85^{ab}	4.52±3.14^{c}	9.27±2.91^{bc}	12.15±10.56^{bc}
Butyrate	1608	107-92-6	RI, MS	2.76±0.80^{cd}	1.31±0.20^{cd}	1.61±1.53^{cd}	5.96±1.42^{bc}	0.77±0.26^{d}	3.52±1.93^{cd}	11.69±4.08^{a}	5.04±0.58^{bcd}	8.45±4.93^{ab}
Caproic acid	1823.1	142-62-1	RI, MS	6.11±0.93^{b}	2.65±0.73^{c}	3.19±0.61^{c}	3.04±0.69^{c}	0.95±0.28^{c}	0.50±0.15^{c}	-	12.25±2.07^{a}	11.4±3.04^{a}
Octanoic acid	2036.1	124-07-2	RI, MS	0.76±0.07^{b}	0.10±0.02^{b}	0.09±0.01^{b}	0.87±0.05^{b}	0.83±0.27^{b}	0.28±0.00^{b}	8.31±2.49^{a}	7.60±1.68^{a}	7.04±2.58^{a}
Decanoic acid	2240.1	334-48-5	RI, MS	0.12±0.07^{c}	0.07±0.02^{c}	0.26±0.10^{c}	1.21±0.44^{bc}	2.10±0.15^{abc}	0.38±0.27^{c}	3.32±2.97^{ab}	4.03±1.22^{a}	3.77±1.42^{a}
2-Methylpropionic acid	1575.4	79-31-2	RI, MS	3.98±0.97^{abc}	5.03±1.46^{abc}	5.41±1.19^{abc}	12.60±1.13^{a}	1.25±0.35^{c}	3.59±1.98^{bc}	10.97±10.66^{ab}	-	7.20±6.56^{abc}
3-Methylbutanoic acid	1649.5	503-74-2	RI, MS	10.14±2.16^{a}	11.90±5.29^{a}	10.02±0.96^{a}	-	1.73±0.56^{b}	3.59±1.97^{b}	0.02±0.01^{b}	-	-
Propionic acid	1561.6	1979-9-4	RI, MS	0.07±0.12	0.99±1.58	-	1.20±0.43	0.16±0.03	0.76±0.23	0.40±0.17	-	-
Phenylacetic acid	2472.8	103-82-2	RI, MS	-	0.01±0.00^{b}	0.05±0.02^{b}	0.80±0.35^{a}	0.47±0.33^{ab}	-	-	-	-
4-Methylvaleric acid	1781.2	646-07-1	RI, MS	-	0.03±0.01^{ab}	0.03±0.01^{ab}	-	0.05±0.01^{ab}	-	0.05±0.03^{ab}	0.03±0.01^{b}	0.07±0.03^{a}
Heptanic acid	1929.9	111-14-8	RI, MS	0.12±0.01	-	-	0.27±0.01	0.39±0.28	-	0.52±0.43	-	-
Valeric acid	2140.4	109-52-4	RI, MS	-	0.03±0.01^{c}	-	0.73±0.14^{c}	0.28±0.11^{a}	0.08±0.06^{b}	-	0.00±0.00^{c}	-
Formic acid	1117.6	64-18-6	RI, MS	-	-	0.03±0.03	0.65±0.66	-	-	-	-	-
Nonanoic acid	2045.1	112-05-0	RI, MS	-	-	0.03±0.01^{c}	0.25±0.09^{b}	0.41±0.08^{a}	-	0.01±0.01^{c}	-	0.01±0.00^{c}

9-Decenoic acid	2196.1	14436-32-9	RI, MS	-	-	0.02±0.00	-	-	-			
Dodecanoic acid	2277.7	143-07-7	RI, MS	-	-	0.02±0.02^{c}	1.07±0.42^{b}	2.00±0.47^{a}	0.34±0.09^{c}	-	-	0.18±0.18^{c}
3-Methylvaleric acid	1718.8	105-43-1	RI	-	-	0.01±0.00	0.02±0.02	0.03±0.02	-	-	-	-
3-Methyl-2-butenoic acid	1595.8	541-47-9	RI, MS	-	-	0.01±0.01	-	-	-	0.02±0.01	0.01±0.01	-
Oxalate	1130.5	144-62-7	RI	-	-	-	0.01±0.01	0.01±0.02	0.03±0.02	-	-	-
Tridecanoic acid	2468.2	638-53-9	RI, MS	-	-	-	0.09±0.01	0.08±0.05	0.03±0.04	-	-	-
Tetradecanoic acid	2580.4	544-63-8	RI, MS	-	-	-	7.99±2.45^{b}	15.58±3.35^{a}	0.22±0.07^{c}	-	-	0.05±0.09^{c}
Pentadecanoic acid	2623.8	1002-84-2	RI, MS	-	-	-	0.06±0.02	0.62±0.21	0.58±0.51			
Hexadecylic acid	2704	1957-10-3	RI, MS	-	-	-	0.55±0.19	0.48±0.12	17.27±24.18	0.29±0.28	-	1.63±2.81
2-Methylacrylic acid	1116.3	79-41-4	RI	-	-	-	0.03±0.04	0.14±0.06	-	-	-	-
2-Acrylic acid	1614.3	1979-10-7	RI	-	-	-	0.06±0.05	0.29±0.03	-	-	-	-
Heptadecanoic acid	2818.8	506-12-7	RI, MS	-	-	-	0.59±0.37	1.29±0.29	-	-	-	-
3-Butyric acid	601.2	2345-51-9	RI	-	-	-	-	0.07±0.04	0.01±0.01	-	-	-
9-Tetraenoic acid	2608.4	544-64-9	RI, MS	-	-	-	-	0.54±0.27	0.36±0.27	-	-	-
2-Methyl butyric acid	1637.8	116-53-0	RI, MS	-	-	-	8.44±2.98	-	-	-	4.99±1.32	5.29±4.65
Benzoic acid	2345.7	65-85-0	RI, MS	-	-	-	0.90±0.99	-	-	-	-	-
Octadecanoic acid	2896.1	1957-11-4	RI, MS	-	-	-	12.75±4.57^{a}	-	-	0.02±0.02^{b}	-	0.34±0.31^{b}
3-Methylvaleric acid	1755.7	105-43-1	RI	-	-	-	-	0.03±0.02^{b}	-	0.08±0.04^{a}	0.04±0.01^{ab}	0.07±0.02^{ab}
3-Decenoic acid	2274.6	15469-77-9	RI	-	-	-	-	0.27±0.29	-	-	-	-
Undecanonic acid	2365.1	112-37-8	RI, MS	-	-	-	-	8.77±12.15	-	-	-	-
Linoleic acid	2859.6	60-33-3	RI, MS	-	-	-	-	3.15±0.63	-	-	-	-
2-Ethyl-hexanoic acid	2042	149-57-5	RI, MS	-	-	-	-	-	0.07±0.01	-	-	0.00±0.00
2-Methyl-4-pentenoic acid	2459.8	1575-74-2	RI	-	-	-	-	-	0.26±0.12	-	-	-

Cis-9-hexadecenoic acid	2899.3	373-49-9	RI, MS	-	-	-	-	-	12.06±5.00	-	-	-
2-Octenoic acid	2153.6	1470-50-4	RI	-	-	-	-	-	-	0.07±0.08	-	-
2-Methyl-2-butenoic acid	2376.2	13201-46-2	RI	-	-	-	-	-	-	0.02±0.01	-	-
3-Butenoic acid	1731.2	625-38-7	RI	-	-	-	-	-	-	-	0.00±0.00	-
4-Hexenoic acid	1938.7	35194-36-6	RI, MS	-	-	-	-	-	-	-	-	0.01±0.01
4-Pentenoic acid	1757.3	591-80-0	RI	-	-	-	-	-	-	-	-	0.06±0.02
Ethyl-9,12-octadienoic acid	2454.9	7619-8-1	RI, MS	-	-	-	-	0.77±0.21	-	-	-	-
				Esters								
Methyl hexanoate	1174.7	106-70-7	RI, MS	2.75±2.10^{a}	1.11±1.44^{ab}	1.12±0.83^{ab}	-	-	-	-	-	-
Ethyl hexanoate	1246.2	123-66-0	RI, MS	0.50±0.56^{a}	0.21±0.14^{ab}	0.16±0.03^{ab}	-	-	0.00±0.00^{b}	0.00±0.00^{b}	0.02±0.01^{b}	0.01±0.00^{b}
Methyl octanoate	1479.9	111-11-5	RI, MS	0.73±0.45	0.42±0.46	0.55±0.29	-	-	-	-	-	-
Ethyl caproate	1513.7	106-32-1	RI, MS	0.15±0.09	0.11±0.06	0.11±0.03	-	-	-	0.02±0.01	0.05±0.02	0.24±0.33
Methyl decanoate	1592.7	110-42-9	RI, MS	0.09±0.04^{ab}	0.11±0.10^{a}	0.13±0.01^{a}	-	-	-	-	-	0.01±0.00^{b}
Ethyl decanoate	1629.8	110-38-3	RI, MS	0.03±0.02^{b}	0.06±0.03^{b}	0.11±0.06^{a}	-	0.01±0.01^{b}	-	0.01±0.00^{b}	0.01±0.01^{b}	0.01±0.00^{b}
Dehydropropionolactone	1990.4	2381-87-5	RI, MS	0.02±0.01^{c}	0.02±0.01^{c}	0.09±0.02^{bc}	0.05±0.03^{c}	-	0.01±0.01^{c}	0.57±0.50^{b}	0.58±0.20^{b}	1.51±0.55^{a}
Methyl isovalerate	1010.9	556-24-1	RI, MS	2.59±1.59	1.54±0.79	1.53±1.52	-	-	-	-	-	-
Methyl phenylacetate	1737	101-41-7	RI	0.07±0.04	0.05±0.03	0.18±0.18	-	-	-	-	-	-
Panthenolide	2000	599-04-2	RI, MS	-	0.35±0.06^{b}	2.16±0.56^{a}	-	0.17±0.03^{b}	-	-	-	-
Methyl butyrate	924	623-42-7	RI, MS	0.64±0.26	-	0.59±0.75	-	-	-	-	-	-
Ethyl phenylpropionate	1862.3	2021-28-5	RI, MS	0.03±0.01	0.01±0.00	-	-	-	-	-	-	-
Ethyl acetate	686.1	141-78-6	RI, MS	-	0.67±0.22	0.59±0.18	-	-	-	-	-	-
Ethyl phenylacetate	1764.2	101-97-3	RI, MS	-	0.02±0.00	0.03±0.01	-	-	-	-	0.03±0.01	0.04±0.01
Ethyl dodecanoate	1835	106-33-2	RI, MS	-	0.00±0.00	0.01±0.00	-	-	-	-	-	0.07±0.06

Propyl acetate	890	109-60-4	RI, MS		5.93±2.28^{a}	-	-	0.01±0.02^{b}	0.26±0.21^{b}	—	0.56±0.23^{b}	0.44±0.25^{b}
Methyl-2-methylbutyrate	1002.2	868-57-5	RI	-	0.45±0.25	-	-	-	-	-	-	-
Butyl acetate	1060.6	123-86-4	RI, MS	-	0.59±0.13^{a}	-	-	-	-	0.02±0.01^{b}	0.04±0.01^{b}	0.04±0.02^{b}
Methyl nonanoate	1542.2	1731-84-6	RI, MS	-	0.01±0.01	-	-	-	-	-	-	-
Ethyl-2-hydroxypropionate	2629.5	97-64-3	RI	-	0.00±0.00	-	-	0.00±0.00	0.02±0.02	-	-	-
Butyrolactone	1584.3	96-48-0	RI, MS	-	-	3.63±0.87^{b}	-	0.67±0.49^{b}	2.06±1.14^{b}	-	7.83±3.63^{a}	-
Methyl valerate	1071.5	624-24-8	RI, MS	-	-	0.05±0.02	-	-	-	-	-	-
Isobutyl acetate	1058.7	110-19-0	RI, MS	-	-	0.31±0.11^{a}	0.02±0.02^{b}	0.02±0.03^{b}	-	-	-	-
Methyl-2-methylbutyrate	1002.1	868-57-5	RI	-	-	0.92±1.09	-	-	-	-	-	-
2-Hydroxy-γ-butyrolactone	2130.3	19444-84-9	RI, MS	-	-	0.00±0.00	-	0.08±0.01	-	-	-	-
Ethyl tetradecanoate	2021.3	124-06-1	RI, MS	-	-	-	0.02±0.02	0.05±0.01	0.03±0.03	-	-	-
Ethyl octadecanoate	2402.1	111-61-5	RI, MS	-	-	-	0.14±0.11^{a}	0.16±0.01^{a}	0.02±0.02^{ab}	-	-	0.00±0.00^{b}
Ethyl linoleate	2454.6	544-35-4	RI, MS	-	-	-	0.15±0.08	-	0.03±0.02			
Methyl tetradecanoate	1980.2	124-10-7	RI, MS	-	-	-	0.03±0.01	0.02±0.00	-	-	-	0.01±0.00
Methyl hexadecate	2181.7	112-39-0	RI, MS	-	-	-	0.23±0.06^{ab}	0.26±0.09^{a}	-	0.07±0.03^{c}	0.11±0.07^{bc}	0.08±0.04^{c}
Ethyl-9-hexdecanoate	2239.4	54546-22-4	RI, MS	-	-	-	0.05±0.04	0.14±0.10	-	-	-	-
Methyl formate	2924	107-31-3	RI	-	-	-	-	0.65±0.47	0.01±0.01	-	-	-
Ethyl palmitate	2218.6	628-97-7	RI, MS	-	-	-	-	0.30±0.22^{a}	0.06±0.04^{b}	0.01±0.00^{b}	0.02±0.03^{b}	0.04±0.01^{b}
Methyl acetate	1350	79-20-9	RI, MS	-	-	-	-	0.15±0.11	0.00±0.00	-	-	-
Ethyl butyrate	1062.7	4341-76-8	RI	-	-	-	-	0.00±0.00	-	-	-	-
Octyl hexadecate	2616.9	16958-85-3	RI	-	-	-	-	0.17±0.14	-	-	-	-
Methyl oleate	2384.4	112-62-9	RI, MS	-	-	-	-	0.34±0.12	-	-	-	-
Ethyl oleate	2416.3	111-62-6	RI, MS	-	-	-	-	0.51±0.14	-	-	-	-

Cyclopentyl-4-ethylbenzoate	1742.9	14779-78-3	RI, MS	-	-	-	-	4.93±3.18	-	-	-	-
2-Methyl acrylate	880.4	922-67-8	RI	-	-	-	-	-	0.01±0.01	-	-	-
2-Vinyl acrylate	1149.4	2177-18-6	RI	-	-	-	-	-	0.01±0.01	-	-	-
Vinyl formate	1261.1	692-45-5	RI	-	-	-	-	-	0.27±0.38	-	-	-
Butyl valerate	1553.7	591-68-4	RI, MS	-	-	-	-	-	0.00±0.00	-	-	-
Heptyl formate	1563	112-23-2	RI	-	-	-	-	-	0.65±0.56	-	-	-
Butyl-2-butenoate	1573.2	7299-91-4	RI, MS	-	-	-	-	-	0.02±0.02	-	-	-
Ethyl-dodecanoate	1700.3	106-33-2	RI, MS	-	-	-	-	-	0.00±0.00	-	-	-
Triethyl phosphate	1741.9	78-40-0	RI, MS	-	-	-	-	-	0.02±0.01	-	-	-
Methyl-2-hydroxy-2-methyl-propionate	1967.4	2110-78-3	RI	-	-	-	-	-	0.03±0.04	-	-	-
Isopropyl myristate	2124.9	110-27-0	RI, MS	-	-	-	-	-	0.07±0.07	-	-	-
Diethyl-1,4-phthalate	2418	636-09-9	RI	-	-	-	-	-	0.16±0.14	-	-	-
Diisobutyl phthalate	2581.6	84-69-5	RI, MS	-	-	-	-	-	0.02±0.02	-	-	-
Dibutyl phthalate	2706.9	84-74-2	RI, MS	-	-	-	-	-	0.08±0.06	-	-	0.96±0.56
Ethyl-p-hydroxybenzoate	2915.7	120-47-8	RI, MS	-	-	-	-	-	0.03±0.02			
Decyl decanoate	2429.8	1654-86-0	RI, MS	-	-	-	-	-	-	0.10±0.03	0.10±0.05	0.05±0.03
Ethyl-2-methyl butyrate	1054.3	7452-79-1	RI, MS	-	-	-	-	-	-	0.02±0.02	0.03±0.03	0.07±0.03
Ethyl-3-methyl butyrate	1066.6	108-64-5	RI, MS	-	-	-	-	-	-	0.01±0.01	0.01±0.00	0.02±0.01
Dimethyl phthalate	2200.6	131-11-3	RI, MS	-	-	-	-	-	-	0.05±0.01	0.04±0.02	0.04±0.02
Diethyl phthalate	2265.8	84-66-2	RI, MS	-	-	-	-	-	-	0.03±0.01	0.04±0.03	0.04±0.01
2-Hydroxy-γ- Butyrolactone	2077.7	19444-84-9	RI, MS	-	-	-	-	-	-	0.03±0.02^{b}	0.04±0.02^{b}	0.15±0.06^{a}
Butyl butyrate	1188	109-21-7	RI, MS	-	-	-	-	-	-	0.00±0.00	0.00±0.00	-
Butyl 2-butenoate	1194.6	7299-91-4	RI, MS	-	-	-	-	-	-	0.00±0.00	0.00±0.00	-

Ethyl heptanate	1378.1	106-30-9	RI, MS	-	-	-	-	-	-	-	0.00±0.00	0.00±0.00
Dimethyl adipate	1745.7	627-93-0	RI, MS	-	-	-	-	-	-	-		
Ethyl-2-hydroxypropionate	1389.6	97-64-3	RI, MS	-	-	-	-	-	-	-	0.00±0.00	0.00±0.00
Methyl acetate	1198.2	79-20-9	RI, MS	-	-	-	-	-	-	0.02±0.02	-	-
Ethyl-4-ethoxybenzoate	2082.2	23676-09-7	RI, MS	-	-	-	-	-	-	0.06±0.04	-	-
Ethyl tetradecanoate	1972.1	124-06-1	RI, MS	-	-	-	-	-	-	0.02±0.01	-	-
Ethyl propionate	1105.5	105-37-3	RI, MS	-	-	-	-	-	-	-	0.00±0.00	-
Methyl heptanate	1008.7	106-73-0	RI, MS	-	-	-	-	-	-	-	0.00±0.00	-
Butyl-3-methyl butyrate	1009.8	106-27-4	RI, MS	-	-	-	-	-	-	-	0.02±0.00	-
Ethyl valerate	1119.3	539-82-2	RI, MS	-	-	-	-	-	-	-	-	0.00±0.00
Methyl-2-butyrate	1018.2	623-43-8	RI, MS	-	-	-	-	-	-	-	-	0.01±0.01
Butyl-2-methyl butyrate	1153.5	15706-73-7	RI, MS	-	-	-	-	-	-	-	-	0.00±0.00
Ethyl-2-methylvalerate	1764.1	39255-32-8	RI, MS	-	-	-	-	-	-	-	-	0.03±0.01
						Alkane						
Pentane	494	109-66-0	RI	0.04±0.04	0.05±0.04	0.09±0.01	0.06±0.05	0.18±0.26	0.03±0.04	0.05±0.01	0.03±0.01	0.03±0.00
N-hexane	505.2	110-54-3	RI	0.01±0.01^{b}	0.02±0.00^{b}	0.01±0.01^{b}	-	-	-	34.46±17.91^{a}	32.53±10.52^{a}	16.18±4.26^{b}
Decane	999.6	124-18-5	RI	4.25±1.05^{a}	2.52±0.73^{b}	1.57±0.25^{b}	-	0.03±0.04^{c}	0.39±0.56^{c}	-	-	0.24±0.11^{c}
Undecanone	1023.9	1120-21-4	RI	0.10±0.04	0.08±0.03	0.02±0.02	-	-	0.02±0.03	-	-	-
Dodecane	1194.2	112-40-3	RI	2.36±1.50^{a}	2.69±1.98^{a}	0.24±0.04^{b}	-	-	0.01±0.01^{b}	0.11±0.05^{b}	0.18±0.05^{b}	0.04±0.02^{b}
Tridecane	1054.1	629-50-5	RI	0.26±0.03^{a}	0.13±0.06^{b}	0.13±0.06^{b}	-	-	-	0.02±0.01^{c}	0.02±0.01^{c}	0.02±0.01^{c}
Tetradecane	1499.9	629-59-4	RI	0.62±0.22^{a}	0.40±0.04^{a}	0.44±0.17^{a}	-	-	-	0.02±0.01^{b}	0.13±0.03^{b}	0.17±0.12^{b}
Cetane	1599.7	544-76-3	RI	0.04±0.04^{ab}	0.01±0.01^{b}	0.04±0.01^{ab}	0.05±0.05^{ab}	0.06±0.06^{ab}	0.06±0.08^{ab}	0.14±0.08^{ab}	0.17±0.11^{a}	-
3-Methylheptane	565.6	589-81-1	RI	0.12±0.11	0.12±0.05	0.09±0.01	-	-	-	-	-	-

3-Methylundecanone	1159.8	1002-43-3	RI	1.35±0.79	1.35±1.10	0.08±0.02	-	-	-	-	-	-
5-Methylnonane	841.8	15869-85-9	RI	0.09±0.01^{a}	0.05±0.00^{b}	0.04±0.01^{b}	-	-	-	-	-	-
4-Methyltetradecane	1462	25117-24-2	RI	0.06±0.01	0.05±0.02	0.06±0.01	-	-	-	-	-	-
3-Methyltridecane	1467.8	6418-41-3	RI, MS	0.48±0.07^{a}	0.21±0.07^{b}	0.28±0.06^{b}	-	-	-	-	0.02±0.01^{c}	-
4-Ethyloctane	829.5	15869-86-0	RI	0.14±0.01	0.11±0.04	-	-	-	-	-	-	-
3-Methylpentadecane	1584	2882-96-4	RI	0.06±0.01	0.04±0.02	-	-	-	-	-	-	-
Octane	585.8	111-65-9	RI	-	0.31±0.08	0.21±0.04	0.47±0.36	-	0.07±0.08	-	-	-
4-Ethyldecane	1134.1	1636-44-8	RI	0.13±0.02	-	-	-	-	-	-	-	-
2,5-Dimethyldecane	1050.2	17312-50-4	RI	0.45±0.40	-	-	-	-	-	-	-	-
5-Methylundecanone	1141.9	1632-70-8	RI	0.37±0.23	-	-	-	-	-	-	-	-
2-Methylcetane	1565.3	1560-92-5	RI	0.01±0.01	-	-	-	-	-	-	-	-
4-Ethylheptane	1031.1	2216-32-2	RI	-	0.19±0.18	-	-	-	-	-	-	-
5-Propyldecane	1132.5	17312-62-8	RI	-	0.14±0.04	-	-	-	-	-	-	-
4-Methyldodecane	1156.6	6117-97-1	RI	-	0.09±0.10	-	-	-	-	-	-	-
3-Methyloctane	1098.5	2216-33-3	RI	-	-	0.22±0.21	-	-	-	-	-	-
5-Methyldecane	1044.4	13151-35-4	RI	-	-	0.30±0.07	-	-	-	-	-	-
3,3-Dimethyloctane	1043.8	4110-44-5	RI	-	-	0.08±0.02	-	-	-	-	-	-
2,2-Dimethylbutane	1057	75-83-2	RI, MS	-	-	0.09±0.13	-	-	-	-	-	-
4-Ethyltetradecane	1576.5	55045-14-2	RI	-	-	0.03±0.00	-	-	-	-	-	-
2,5-Dimethyltridecane	1400.3	56292-66-1	RI	-	-	0.11±0.03	-	-	-	-	-	-
2,3,5-Trimethylheptane	1102.4	20278-85-7	RI, MS	-	-	0.33±0.07	-	-	-	-	-	-
Butane	670.9	106-97-8	RI	-	-	-	-	0.01±0.01	0.01±0.01	-	-	-

Heptane	517.4	142-82-5	RI	-	-	-	0.12±0.14[b]	-	0.02±0.03[b]	1.09±0.85[a]	0.21±0.28[ab]	0.80±0.70[ab]
Propane	719.1	74-98-6	RI	-	-	-	0.28±0.33	0.00±0.00	-	-	-	-
3-Methyl-heptadecane	1754	6418-44-6	RI, MS	-	-	-	0.01±0.01	0.03±0.03	-	-	-	-
2,3-Dimethylbutane	716.2	79-29-8	RI	-	-	-	-	0.18±0.25	-	-	-	-
3-Methyl-heptane	612.5	589-81-1	RI	-	-	-	-	-	0.10±0.10	-	-	-
2-Nitro-propane	783	79-46-9	RI	-	-	-	-	-	0.00±0.00	-	-	-
Neopentane	1486.7	463-82-1	RI	-	-	-	-	-	0.02±0.02	-	-	-
4-Propyl-heptane	2821.1	55044-10-5	RI	-	-	-	-	-	0.02±0.03	-	-	-
Heptadecane	1634.5	629-78-7	RI	-	-	-	-	-	-	0.06±0.03	0.06±0.03	0.03±0.01
Octadecane	1731.4	593-45-3	RI	-	-	-	-	-	-	0.04±0.01		
2-Methyltridecane	1410.7	1560-96-9	RI	-	-	-	-	-	-	-	0.00±0.00	0.01±0.00
2-Undecanone	1569.3	112-12-9	RI, MS	-	-	-	-	-	-	-	0.01±0.00	-
4-Methyldecane	1085.8	2847-72-5	RI	-	-	-	-	-	-	-	-	0.01±0.00
4-Methylundecanone	1136.2	2980-69-0	RI, MS	-	-	-	-	-	-	-	-	0.01±0.00
3-Methylheptadecane	1701.2	6418-44-6	RI, MS	-	-	-	-	-	-	-	-	0.01±0.00
						Olefin						
1-Decene	1035.6	872-05-9	RI, MS	0.44±0.07[a]	-	0.14±0.04[b]	-	-	-	0.00±0.00[c]	0.01±0.00[c]	0.01±0.01[c]
2-Octene	1283.4	111-67-1	RI, MS	0.62±0.58	-	-	-	-	-	-	-	-
5-Methyl-2-heptene	1760	22487-87-2	RI	0.02±0.00	-	-	-	-	-	-	-	-
3,5,5-Trimethyl-1-hexene	549.7	4316-65-8	RI	0.00±0.00	-	-	-	-	-	-	-	-
3-Methyl-3-heptene	578.5	7300-03-0	RI	-	-	0.01±0.01	-	-	-	-	-	-
1,3-Octadiene	1486.2	1002-33-1	RI, MS	-	-	0.03±0.01	-	-	-	-	-	-
Propylene	1150.7	115-07-1	RI	-	-	-	0.01±0.01	0.42±0.56	7.4±10.44	-	-	-

1-Heptene	535.9	592-76-7	RI, MS	-	-	-	0.06±0.06	-	-	-	-	-
1-Pentene	1324.6	109-67-1	RI	-	-	-	0.07±0.10	-	-	-	-	-
1-Hexene	1451.5	592-41-6	RI	-	-	-	0.05±0.07	-	-	-	-	-
7-Methyl-1-octene	1688.1	13151-06-9	RI	-	-	-	-	-	0.00±0.01	-	-	-
9-Octadecene	2436.8	5090-41-5	RI	-	-	-	-	-	0.15±0.09	-	-	-
1-Dodecene	1219.5	112-41-4	RI, MS	-	-	-	-	-	-	0.01±0.00	0.01±0.00	0.01±0.01
2-Methyl-1-hexene	1030.2	6094-2-6	RI	-	-	-	-	-	-	0.01±0.01	-	-
2,4-Dimethyl-2-pentene	693.3	625-65-0	RI	-	-	-	-	-	-	0.07±0.01	-	-
3-Heptane	1643.3	592-78-9	RI	-	-	-	-	-	-	0.07±0.03	-	-
1-Tetradecene	1509	1120-36-1	RI, MS	-	-	-	-	-	-	-	0.00±0.00	-
6-Methyl-1-octene	1181.8	13151-10-5	RI	-	-	-	-	-	-	-	0.01±0.01	-
5-Dodecene	1267.3	7206-28-2	RI	-	-	-	-	-	-	-	-	0.00±0.00
2,2-Dimethyl-3-octene	1000.4	86869-76-3	RI	-	-	-	-	-	-	-	-	0.02±0.01
						Pyrazine						
Trimethyl pyrazine	1507.7	14667-55-1	RI, MS	1.29±0.89^{b}	-	2.68±0.81^{a}	0.01±0.01^{c}	0.44±0.32^{c}	0.23±0.14^{c}	0.23±0.14^{c}	0.13±0.05^{c}	0.68±0.20^{bc}
2,3,5,6-Tetramethylpyrazine	1532.2	1124-11-4	RI, MS	0.46±0.65	-	0.60±0.53	-	-	0.10±0.07	0.07±0.04	0.04±0.04	0.31±0.40
2,3-Dimethylpyrazine	1434.9	5910-89-4	RI, MS	0.43±0.25^{a}	-	0.21±0.06^{b}	-	-	0.03±0.03^{b}	0.04±0.02^{b}	0.01±0.01^{b}	0.04±0.02^{b}
2,6-Diethylpyrazine	1516.6	13067-27-1	RI, MS	-	-	0.09±0.08^{a}	-	0.01±0.01^{b}	0.01±0.01^{b}	0.00±0.00^{b}	0.00±0.00^{b}	0.00±0.00^{b}
2-Methylpyrazine	1314.6	109-08-0	RI, MS	0.29±0.26	-	-	-	-	0.03±0.04	-	-	-
2,6-Dimethylpyrazine	1417	108-50-9	RI, MS	0.61±0.58^{ab}	-	2.91±2.58^{a}	-	-	0.14±0.10^{b}	-	0.10±0.09^{b}	0.83±0.86^{ab}
2-Ethyl-6-methyl-pyrazine	1493.5	13925-03-6	RI, MS	-	-	0.20±0.03^{a}	-	-	-	0.01±0.01^{b}	0.01±0.00^{b}	0.04±0.02^{b}
Methyl pyrazine	1281.4	109-08-0	RI, MS	-	-	0.25±0.22^{b}	-	-	-	0.05±0.03^{b}	0.03±0.01^{b}	0.06±0.03^{a}
Vinyl pyrazine	1551.3	4177-16-6	RI, MS	-	-	-	-	-	0.13±0.18	-	-	-

Ethyl pyrazine	2172.9	13925-00-3	RI, MS	-	-	-	-	-	0.07±0.09	-	-	0.83±0.86
2,5-Dimethylpyrazine	1354.5	123-32-0	RI, MS	-	-	-	-	-	-	0.40±0.18	0.00±0.00	-
Pyrazine	1865.9	290-37-9	RI, MS	-	-	-	-	-	-	-	0.00±0.00	-
2-Acetyl-3-methylpyrazine	1623.8	23787-80-6	RI, MS	-	-	-	-	-	-	-	-	0.00±0.00
2,3-Dimethyl-5-ethyl pyrazine	1504	15707-34-3	RI, MS	-	-	-	-	-	-	-	-	0.02±0.01
2,3,5-Trimethyl-6-ethylpyrazine	1530.5	17398-16-2	RI, MS	-	-	-	-	-	-	-	-	0.01±0.01
						Furan						
2-Pentylfuran	1232.8	3777-69-3	RI, MS	0.22±0.21[ab]	0.34±0.18[ab]	0.58±0.49[a]	0.26±0.22[ab]	0.5±0.26[ab]	0.43±0.38[ab]	0.04±0.03[ab]	0.02±0.01[b]	0.08±0.02[ab]
2-Ethyl-furan	820.3	3208-16-0	RI, MS	-	0.04±0.05	-	-	-	-	-	-	-
5-Methyl-2-acetylfuran	1590.7	1193-79-9	RI, MS	-	0.01±0.01	-	-	-	-	-	-	-
2-Propyl-furan	1515.9	4229-91-8	RI, MS	-	-	-	0.01±0.01	0.01±0.01	-	0.01±0.01	0.01±0.01	-
2-Hexyl-furan	1581	3777-70-6	RI, MS	-	-	-	0.03±0.04	-	0.01±0.01	-	-	-
2-Methylfuran	2105.1	534-22-5	RI, MS	-	-	-	0.00±0.00	-	-	-	-	-
Furan	1086.8	110-00-9	RI, MS	-	-	-	-	-	0.05±0.06	0.00±0.00	-	-
2-Ethyl-5-methyl-furan	1584.9	1703-52-2	RI	-	-	-	-	-	0.01±0.01	-	-	-
2,5-Dibutylfuran	1161	72636-53-4	RI	-	-	-	-	-	-	0.00±0.00	0.00±0.00	0.00±0.00
3-Methyl-furan	1470.8	930-27-8	RI, MS	-	-	-	-	-	-	0.00±0.00	-	-
						Sulfide						
Dimethyl trisulfide	1455.4	3658-80-8	RI, MS	0.02±0.02[b]	0.01±0.00[b]	0.01±0.00[b]	1.21±0.99[a]	0.65±0.10[ab]	0.34±0.19[b]	0.00±0.00[b]	-	0.00±0.00[b]
Dimethyl sulfide	557.9	75-18-3	RI, MS	-	-	0.05±0.00[a]	-	0.01±0.01[b]	-	0.00±0.00[b]	-	-
Dimethyl disulfide	1048.4	624-92-0	RI, MS	-	-	-	0.95±1.34	1.71±2.39	-	-	-	-
Dimethyl tetrasulfide	1693.9	5756-24-1	RI, MS	-	-	-	-	0.03±0.02	-	-	-	-
Methyl mercaptan	510.6	74-93-1	RI, MS	-	-	-	0.38±0.28	0.16±0.12	-	-	-	-

Notes: “-“ indicates that the substance is not detected whiles “0.00” indicates that the value is less than 0.01. All the value is expressed as the percentage of peak area (%). The relative percentage is the ratio of the volatile component to the total volatile aroma compounds in one aging time.

[1] Volatile component were identified by RI and MS

[2] Retention index, calculated according to peak time of series alkanes under the same gas phase conditions

[a, b, c, d] Means with different letters with in a row differ significantly ($P < 0.05$)
